# Identification of Blood Let-7e-5p as a Biomarker for Ischemic Stroke

**DOI:** 10.1371/journal.pone.0163951

**Published:** 2016-10-24

**Authors:** Suli Huang, Ziquan Lv, Yi Guo, Lu Li, Yanwei Zhang, Li Zhou, Binyao Yang, Shuang Wu, Ying Zhang, Changhui Xie, Shanshan Li, Jinquan Cheng

**Affiliations:** 1 Shenzhen Center for Disease Control and Prevention, Shenzhen, Guangdong, China; 2 Department of Neurology, People’s Hospital of Shenzen, Guangdong, China; 3 Key Laboratory of Environment and Health, Ministry of Education and Ministry of Environmental Protection, and State Key Laboratory of Environmental Health (Incubating), School of Public Health, Tongji Medical College, Huazhong University of Science and Technology, Wuhan, Hubei, China; 4 State Key Lab of Respiratory Disease, Institute for Chemical Carcinogenesis, School of Public Health, Guangzhou Medical University, Guangzhou, Guangdong, China; Kunming University of Science and Technology, CHINA

## Abstract

Circulating microRNAs (miRNAs) are emerging as novel disease biomarkers. Using a miRNA microarray, we previously showed that the whole blood level of let-7e-5p was significantly higher in ischemic stroke patients than in control subjects. However, the association between let-7e-5p expression and the occurrence of ischemic stroke remains unknown. In this study, we validated the expression levels of let-7e-5p in two case-control populations using miRNA TaqMan assays and further investigated the potential targets of let-7e-5p. The results suggest that the blood level of let-7e-5p was significantly higher in patients with ischemic stroke than in controls (p<0.05). Higher levels of let-7e-5p were associated with increased occurrence of ischemic stroke (adjusted OR, 1.89; 95% CI, 1.61~2.21, p<0.001) in the combined population. The addition of let-7e-5p to traditional risk factors led to an improvement in the area under the curve, which increased from 0.74 (95% CI, 0.70~0.78) to 0.82 (95% CI, 0.78~0.85), with a net reclassification improvement of 16.76% (p<0.0001) and an integrated discrimination improvement of 0.10 (p<0.0001) for patients with ischemic stroke. Bioinformatics prediction and cell experiments suggested that the expression levels of four genes enriched in the MAPK signaling pathway were down-regulated by let-7e-5p transfection. Specifically, the expression levels of the genes CASP3 and NLK were significantly lower in ischemic stroke patients than in controls and were negatively correlated with let-7e-5p expression. In summary, our study suggests the potential use of blood let-7e-5p as a biomarker for ischemic stroke and indicates its involvement in the related pathomechanism.

## Introduction

Globally, stroke is the second leading cause of death in people more than 60 years old, and it is the fifth leading cause of death in people between 15 and 59 years old [1]. In China, as in other countries, ischemic stroke is the most common type of stroke, accounting for 43% to 79% of the total number of strokes [2]. Ischemic stroke has several etiological origins, the most common one of which is the narrowing of the neck or head arteries through atherosclerosis. Atherosclerosis is a chronic disease process involving endothelial dysfunction, lipid disturbance, platelet activation, thrombosis, and inflammation. Multiple risk factors have also been identified for ischemic stroke, including age, male sex, smoking, history of hypertension and diabetes mellitus. However, a diagnosis biomarker for ischemic stroke has not been found. In recent years, advances in epigenomics have promoted the generation of many novel disease diagnosis biomarkers, among which microRNA (miRNA) have attracted extensive interest.

MiRNAs are short, endogenous, and non-coding RNAs that regulate gene expression at the posttranscriptional level by binding to 3-untranslated regions of their target mRNAs [3,4]. miRNAs have emerged as important regulators and fine-tuners of a range of pathophysiological cellular effects and molecular signaling pathways involved in atherosclerosis [5]. Moreover, miRNAs are also involved in the regulation of stroke-related cellular and molecular networks, and the identification of these miRNAs may provide new insights into the underlying mechanisms of stroke. For example, miRNAs are involved in the modulation of cerebral edema [6,7], a common consequence of stroke that contributes to tissue and cellular damage following ischemia. Microarray studies in animal models have also shown that numerous miRNAs are rapidly altered following ischemic preconditioning, which leads to the modulation of several key signaling pathways and an increase in cytoprotection and cellular regeneration in the affected brain tissue [8,9]. Several studies have begun to analyze miRNAs in plasma or serum to identify biomarkers for disease risk prediction or diagnosis [10–12]. However, identifying biomarkers using plasma or serum miRNAs has limitations because of the extremely low concentration of circulating miRNAs. Seeking to circumvent this problem, we hypothesized that miRNAs from peripheral whole blood could also be used. Peripheral blood contains immune cells, which have close contact with the vascular wall, a key component of the inflammatory response and are involved in the development and progression of atherosclerosis. Hoekstra et al. reported the dysregulation of several miRNAs in peripheral blood mononuclear cells from patients with coronary artery disease [13]. Another study revealed that 157 miRNAs were differentially expressed in the whole blood of stroke patients and controls [14]. Ontological analysis predicted that the targets of deregulated miRNAs were involved in angiogenesis, hypoxia, endothelial cell regulation, and immune response. Limited by a small sample size, however, the study discussed above did not determine the association between miRNA expression and stroke. In addition, the functional role of circulating miRNAs in ischemic stroke remains unknown.

In this study, we aimed to determine whether the whole blood level of let-7e-5p is differentially expressed in patients with ischemic stroke. We previously explored the miRNA expression profiles of 25 ischemic stroke patients and 25 control subjects using miRNA microarray and found that 455 miRNAs were differentially expressed (unpublished data, p<0.05). Of the differentially expressed miRNAs, let-7e-5p became our focus after reviewing the literature. For example, Li et al. found that the plasma level of let-7e-5p was significantly higher in essential hypertension patients than in control subjects [15], and let-7e-5p was also involved in the response of macrophages to lipopolysaccharide [16]. Because of the important role of let-7e-5p in the cardiovascular system, we selected let-7e-5p for further investigation and analyzed its potential as a biomarker for ischemic stroke.

## Materials and Methods

### Study design and populations

We determined the blood expression level of let-7e-5p in two independent case-control populations. Ischemic stroke patients from the stage I population were recruited from Shenzhen No. 2 Hospital between April 2013 and December 2013. Patients from the stage II population were consecutively recruited from Shenzhen People’s Hospital between June 2012 and December 2014. All subjects were inpatients diagnosed with ischemic stroke for the first time and were recruited based on the appearance of a new and abrupt focal neurological deficit, with neurological symptoms and signs persisting for more than 24 h, as described previously [17]. Ischemic stroke was confirmed by positive findings on head CT or MRI according to the International Classification of Disease (9th Revision, codes 430 to 438). Patients with a history of stroke, peripheral arterial occlusive disease or cancer were excluded from this study. Subjects without medical history of cerebrovascular diseases were selected as controls during a physical examination at the hospital and were matched with patients by age and sex. Approximately 5 mL of vein blood samples were collected from each participant in EDTA-anticoagulant tubes and stored at −80°C until use. Blood samples from patients with ischemic stroke were collected within 12 h after hospital admission.

For all participants, structured questionnaires were used by trained interviewers to collect information about demographic characteristics and clinical biochemistry. The ethics committee of the Shenzhen Center for Disease Control and Prevention approved this study, and written informed consent was obtained from each participant.

### Total RNA isolation

Approximately 200 μL of whole blood was used from each sample to isolate total RNAs using the *mir*Vana PARIS miRNA Isolation Kit (Ambion 1556, Austin, TX) according to the manufacturer’s protocol but with slight modifications, namely, each sample was treated twice with acid-phenol chloroform. The total RNAs of cultured cells were isolated using Trizol reagent (Invitrogen, AL, USA). To detect the expression levels of genes, the total RNA of each sample was isolated from 500 μL of whole blood using Trizol LS reagent (Invitrogen, AL, USA) according to the manufacturer’s protocol.

### qRT-PCR assays

For miRNA detection, the input RNAs were reverse transcribed (RT) in a small-scale reaction using the TaqMan miRNA Reverse Transcription Kit (Applied BioSystems, Foster City, CA) following the manufacturer’s protocol (5 μL total volume with 1 μL of input RNA; components other than the input RNA were prepared as a large-volume master mix). RT products were diluted 1:5 and subjected to qPCR in triplicate using the TaqMan miRNA Assay Kit (Applied BioSystems, Foster City, CA) according to the manufacturer’s protocol in a small-scale reaction (10 μL total volume with 4.5 μL of diluted RT products; components other than input RT products were prepared as a large-volume master mix). MiRNA expression levels were normalized to U6 and calculated using the equation 2^-ΔCt^, where ΔCt = cycle threshold (Ct) (miRNA)−Ct _(U6)_.

For the detection of gene expression, first strand cDNA was synthesized from equal amounts of RNA using the Super Script ^™^ First-Strand Synthesis System (Life Technologies, USA). Quantitative PCR was performed in triplicate with the 7500 Real-time PCR System (Applied Biosystems, USA) using SYBR Premix Ex Taq^™^ (Takara, Dalian, China). Gene expression values were calculated using the comparative quantitative method (the -ΔΔCT method) and normalized to values obtained from the amplification of beta-actin. PCR amplification was performed using the sets of primers designed by Primer 5.0, and the abbreviation list for the gene names were listed in S1 Table.

### Target gene prediction and pathway analysis

The miRNA target genes were predicted using three bioinformatics tools: TargetScan (http://www.targetscan.org/), miRanda (http://www.microrna.org/microrna/home.do) and Pictar (http://pictar.mdc-berlin.de/). The pathway enrichment of predicted genes was analyzed using the DAVID tool (http://david.abcc.ncifcrf.gov/).

### Cell culture and transfection

The U937 cell line, purchased from the Procell company (Wuhan, China), was cultured in RPMI-1640 medium (GIBCO) supplemented with 10% fetal calf serum (GIBCO) and penicillin/streptomycin in a humidified atmosphere containing 5% CO_2_ at 37°C. Approximately 50 nM let-7e-5p mimic or control mimic purchased from RIBObio (Guangzhou RIBObio company) was transfected into U937 cells for 48 h using the ribo*FECT* CP transfection kit (Guangzhou RIBObio company) according to the manufacturer’s protocol. All experiments were conducted with cells at a logarithmic stage of growth.

### Statistical analysis

The normal distribution of data was tested using the 1-sample Kolmogorov–Smirnov test. Continuous variables were expressed as the mean ± SD or median (25th–75th quartile), and categorical variables were expressed as frequency. Differences in clinical characteristics between cases and controls were examined by the χ^2^ test for categorical variables, Student’s *t* test for normally distributed data, or the Mann–Whitney U test for skewed data. The difference in circulating let-7e-5p expression levels or mRNA levels of predicted genes between cases and controls was examined using Student’s *t* test. Differences in mRNA levels of genes among the transfected cells were analyzed by one-way ANOVA. The odds ratios (ORs) and 95% confidence intervals (CIs) were calculated to assess the association between let-7e-5p and ischemic stroke using the multivariate logistic regression model after adjusting for conventional risk factors, including age, sex, smoking, drinking, hypertension and diabetes mellitus. The ability of let-7e-5p to discriminate patients with ischemic stroke from controls or to reclassify patients with ischemic stroke was determined by the receiver operating characteristic curve and reclassification analysis using 2 models: a baseline model including conventional risk factors, and a model that incorporated the factors from the baseline model and let-7e-5p expression. Correlations between miRNA and gene expression levels in the blood were analyzed using the Pearson correlation test. Correlations between miRNA expression and platelet parameters were analyzed using the Spearman correlation test. All statistical analyses were performed using SPSS 11.0 software (Statistical Package for the Social Sciences, Chicago, IL). A value of p<0.05 was considered significant (2-tailed).

## Results

### General characteristics of the study population

The general characteristics of the two case-control populations are shown in Table 1. For the stage I population, the levels of high-density lipoprotein cholesterol (HDL-c), low-density lipoprotein cholesterol (LDL-c), and platelet distribution width (PDW) were significantly lower than in the controls (p<0.05). The levels of mean platelet volume (MPV), smoking frequency, drinking, and history of hypertension or diabetes mellitus were significantly higher in patients (p<0.05). The other variables showed no significant difference.

**Table 1:** General characteristics of the study population.

Variables	Stage I			Stage II		
	Control (n = 44)	Case (n = 44)	*P* value	Control (n = 302)	Case (n = 302)	*P* value
Age, year	55.18 ± 10.48	57.32 ± 11.35	0.362*	55.87 ± 10.42	57.50 ± 10.40	0.054*
Male, %	61.4	59.1	0.773†	60.1	60.1	1.000†
FG, mmol/L	5.25 (4.78, 5.85)	5.34 (4.66, 8.00)	0.441‡	5.28 (4.93, 5.86)	5.55 (4.98, 6.89)	<0.001‡
TC, mmol/L	4.97 ± 0.63	4.59 ± 1.45	0.129*	5.05 (4.32, 5.62)	5.02 (4.22, 5.88)	0.467‡
TG, mmol/L	1.28 (1.07, 1.59)	1.32 (1.05, 1.86)	0.952‡	1.37 (0.94, 2.28)	1.33 (0.96, 1.84)	0.163‡
HDL-c, mmol/L	1.25 ± 0.30	1.11 ± 0.28	0.038*	1.26 ± 0.31	1.05 ± 0.27	<0.001*
LDL-c, mmol/L	3.01 ± 0.57	2.54 ± 0.83	0.005*	3.00 (2.58, 3.46)	2.89 (2.44, 3.73)	0.540‡
PLT (×10^9^/L)	242 ± 47	220 ± 56	0.052*	228 (197, 262)	219 (176, 266)	0.071‡
PDW, %	16.00 (15.00, 17.00)	14.00 (12.00, 16.18)	0.002‡	16.00 (15.70, 16.40)	16.00 (12.80, 16.60)	0.504‡
MPV, fL	8.73 ± 1.69	10.59 ± 1.24	<0.001*	8.40 (7.55, 9.30)	9.20 (7.80, 10.20)	<0.001‡
PCT	0.21 ± 0.05	0.23 ± 0.05	0.087*	0.19 (0.16, 0.22)	0.19 (0.16, 0.24)	0.306‡
Smoking, %	15.9	43.2	<0.001†	15.9	22.2	0.279†
Drinking, %	11.4	29.5	0.001†	17.2	8.3	0.054†
Hypertension, %	13.6	52.3	<0.001†	20.2	57.0	<0.001†
Diabetes mellitus,%	9.1	29.5	<0.001†	5.6	17.9	0.009†

Data are expressed as mean ± SD, median (25th, 75th quartiles) or percentages. FG, fasting glucose; TC, total cholesterol; TG, triglycerides; HDL-C, high-density lipoprotein cholesterol; LDL-C, low-density lipoprotein cholesterol; PLT, blood platelet; PDW, platelet distribution width; MPV, mean platelet volume; PCT, plateletcrit.

* Student’s *t* test for the difference between ischemic stroke patients and controls.

^†^ Chi-square test for the difference in the distribution frequencies between ischemic stroke patients and controls.

^‡^ Mann–Whitney U test for the differences between ischemic stroke patients and controls.

For the second population, the levels of fasting glucose, MPV, and history of hypertension and diabetes mellitus were significantly higher in patients than in control subjects (p<0.05), whereas the level of HDL-c was significantly lower in patients (p<0.05). The other variables showed no differences between the two groups.

### Association of the whole blood level of let-7e-5p with the occurrence of ischemic stroke

**Fig 1:**
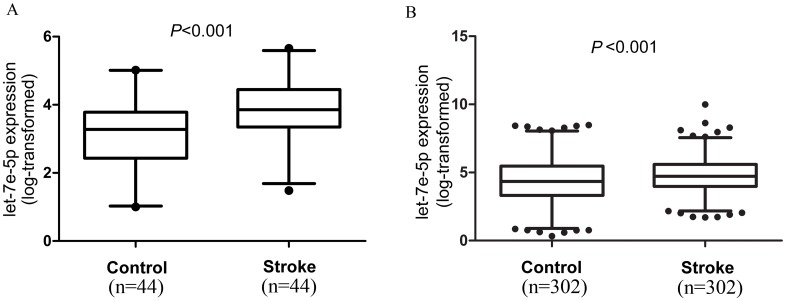
The expression levels of let-7e-5p in stage I and II populations. Let-7e-5p expression was detected in (A) stage I (n = 44 patients and controls) and (B) stage II (n = 302 patients and controls) population. The relative expression levels were normalized to U6 and then log-transformed. The whiskers of the plots represent the 2.5 to 97.5 percentiles.

We detected the whole blood level of let-7e-5p in the first case-control population. As shown in Fig 1, the results suggested that the expression level of let-7e-5p was significantly higher in ischemic stroke patients than in control subjects (Fig 1A, log-transformed expression levels relative to U6, 3.92± 0.74 vs. 3.14±0.98, p<0.001). In the second case-control population, we found that the expression level of let-7e-5p remained significantly higher in ischemic stroke patients than in control subjects (Fig 1B, log-transformed expression levels relative to U6, 4.99±1.04 vs. 4.07±1.37, p<0.001).

Pooled analysis was performed by combining the two populations. The results showed that higher levels of let-7e-5p were associated with an increased risk of ischemic stroke (adjusted OR, 1.89; 95% CI, 1.61~2.21; P<0.001; Table 2) after adjusting for conventional risk factors, including age, sex, smoking, drinking, history of hypertension and diabetes mellitus.

**Table 2:** Association of the expression level of let-7e-5p with ischemic stroke.

Variables	Odds Ratio (95% CI)*	*P* value
age	0.99 (0.97–1.01)	0.265
sex	1.34 (0.90–1.99)	0.152
smoking	2.93 (1.77–4.87)	<0.001
drinking	0.45 (0.25–0.79)	0.006
Hypertension	5.10 (3.47–7.48)	<0.001
Diabetes mellitus	2.36 (1.32–4.22)	0.004
let-7e-5p	1.89 (1.61–2.21)	<0.001

CI, confidence interval.

*By logistic regression analysis.

### Diagnostic value of let-7e-5p by receiver operating characteristic curve and reclassification analysis

The diagnostic value of let-7e-5p was evaluated in the combined population. As shown in Table 3, the area under the receiver operating characteristic curve was 0.74 (95% CI, 0.70~0.78) for the baseline model and significantly increased to 0.82 (95% CI, 0.78~0.85) when let-7e-5p was added to the prediction model (p<0.05). The net reclassification improvement and integrated discrimination improvement were computed to determine the ability of miRNAs to reclassify patients misclassified by the baseline model. let-7e-5p was able to reclassify a significant portion of patients, with a net reclassification improvement (NRI) of 16.76% (95% CI, 11.16%~22.37%; p<0.0001). The integrated discrimination improvement (IDI) for let-7e-5p was 0.10 (95% CI, 0.08~0.13; p<0.0001).

**Table 3:** AUC, NRI, and IDI calculations for let-7e-5p in the combined population.

Variables	let-7e-5p
Baseline model AUC (95% CI)	0.74 (0.70–0.78)
Extended model AUC (95% CI)	0.82 (0.78–0.85)
NRI,% (95% CI)	16.76(11.16–22.37)
IDI (95% CI)	0.10 (0.08–0.13)

AUC calculations are based on a multivariate logistic regression analysis including age, sex, smoking, drinking, hypertension, diabetes mellitus, total cholesterol, and triglycerides (baseline model) or the addition of let-7e-5p (extended model). AUC, area under the receiver operating characteristic curve; CI, confidence interval; IDI, integrated discrimination improvement; and NRI, net reclassification improvement.

### Correlation between let-7e-5p expression and platelet parameters

**Fig 2:**
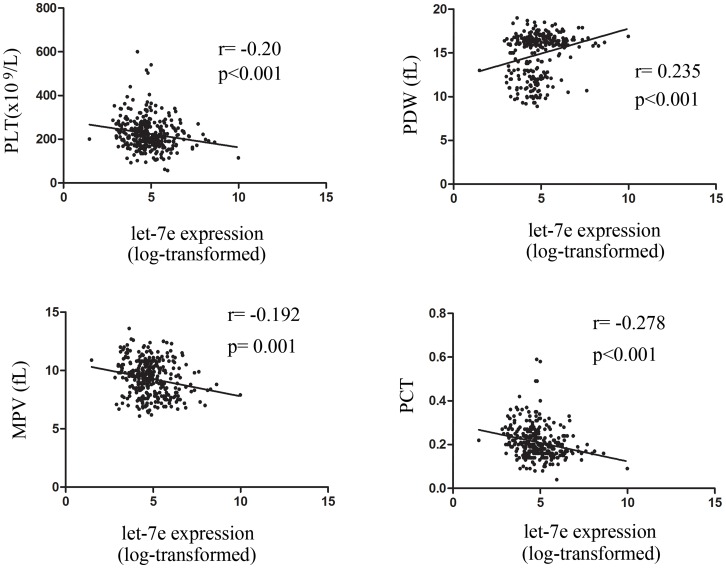
Correlations between let-7e-5p expression and platelet parameters. The correlation was evaluated by the Spearman test in the combined patient population.

Platelet parameters play an important role in the pathogenesis of vascular diseases [18]. We further evaluated the correlation between let-7e-5p expression and platelet parameters in patients from the combined population, assessing platelet count value (PLT), MPV, PDW and plateletcrit (PCT). The results suggested that let-7e-5p was negatively correlated with PLT (r = -0.20, p<0.001), MPV (r = -0.192, p = 0.001) and PCT (r = -0.278, p<0.001). Conversely, let-7e-5p expression was positively correlated with PDW (r = 0.235, p<0.001), as shown in Fig 2.

### Identification of let-7e-5p target genes

Using bioinformatics tools, we found that a total of 1800 genes were predicted to be targets of let-7e-5p. These genes were input into DAVID (http://david.abcc.ncifcrf.gov/) to obtain information about the signaling pathways in which the target genes may enriched. As shown in Table 4, the results suggested that 23 genes were enriched in the MAPK signaling pathway (p<0.001), 13 genes were enriched in the p53 signaling pathway (p = 0.017), 13 genes were enriched in the adherens junction (p = 0.042), and 14 genes were enriched in the TGF-beta signaling pathway (p = 0.047). Because most of the genes were enriched in the MAPK signaling pathway and because the relationship between let-7e-5p and the MAPK signaling pathway remains largely unknown, we selected these 23 genes for further investigation.

**Table 4:** The enriched signal transduction pathways of predicted genes by let-7e-5p.

KEGG Terms	Count of Genes	*P*value	Gene Symbols
MAPK signaling pathway	23	<0.001	FGFR2, PDGFB, CACNB4, ATF2, CASP3, MAP3K2, ELK4, RASGRP1, MAP3K1, PAK1, MAP2K7, PTPN7, NLK, TGFBR1, PTPRR, TP53, CACNG4, CDC25B, MAP4K3, NRAS, MAP4K4, NGF, MRAS
p53 signaling pathway	13	0.017	STEAP3, TP53, IGF1, CHEK1, CCNG2, SESN3, CDKN1A, CASP3, RRM2, RCHY1, MDM4, FAS, THBS1
Adherens junction	13	0.042	PTPRB, TGFBR1, NLK, SMAD2, CDH1, ACP1, VCL, ACVR1C, ACTG1, ACVR1B, IGF1R, INSR, MLLT4
TGF-beta signaling pathway	14	0.047	BMP2, ACVRL1, E2F5, TGFBR1, GDF6, SMAD2, DCN, ACVR1C, ACVR1B, ACVR2B, ZFYVE16, THBS1, CHRD, THBS3

KEGG, Kyoto Encyclopedia of Genes and Genomes; TGF, transforming growth factor.

**Fig 3:**
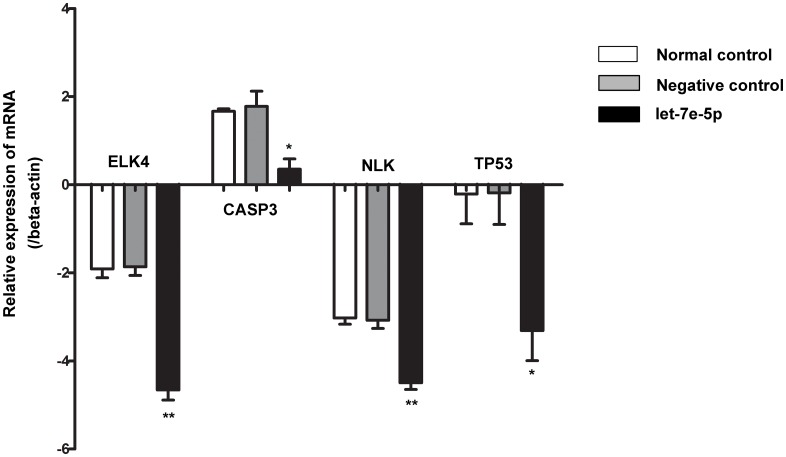
The mRNA levels of the target genes in different cell groups. U937 cells were transfected with 50 nM control mimics (negative control) or 50 nM let-7e-5p mimics. The normal control is normal cultured cells. *p<0.05 compared to the negative control; **p<0.01 compared to the negative control.

To investigate the regulation of genes by let-7e-5p, we transfected the miRNA mimics into U937 cells and detected the changes in gene expression. As shown in Fig 3, the mRNA levels of the ETS-domain protein (ELK4), caspase 3 (CASP3), nemo-like kinase (NLK), and tumor protein p53 (TP53) were all significantly lower in the miRNA-transfected cells than in the control group. No difference was found between the mimic control and control groups for these four genes (p>0.05). The expression levels of the other 19 genes showed no significant differences among the three groups of treated cells (data not shown) (p>0.05).

### Correlations between let-7e-5p and target gene expressions

**Fig 4:**
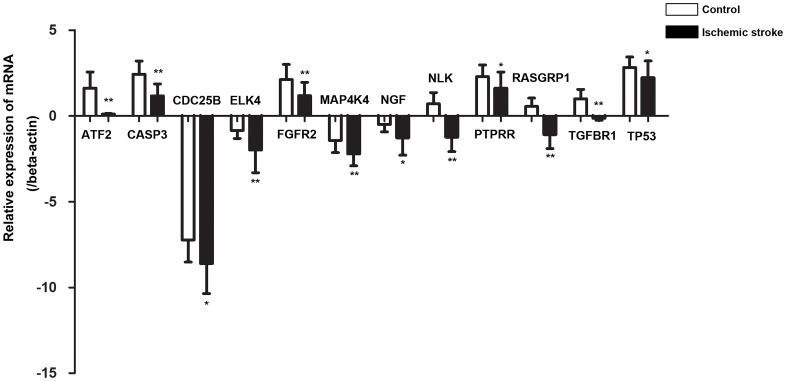
The whole blood mRNA levels of the target genes in control subjects (n = 20) and ischemic stroke patients (n = 20). The relative expression levels were normalized to beta-actin. *p<0.05; **p<0.01.

To further investigate the regulation of genes by let-7e-5p, we detected the expression levels of let-7e-5p and the 23 genes in the whole blood of 20 ischemic stroke patients and 20 control subjects from the second study population. The expression level of let-7e-5p was significantly higher in patients than in control subjects (p = 0.042, shown in S1 Fig), whereas the mRNA levels of 12 genes were found to be significantly lower in the patient group than in the control group (Fig 4, p<0.05). These genes were activating transcription factor 2 (ATF2), CASP3, cell division cycle 25B (CDC25B), ELK4, fibroblast growth factor receptor 2 (FGFR2), mitogen-activated protein kinase kinase kinase kinase 4 (MAP4K4), nerve growth factor (NGF), NLK, protein tyrosine phosphatase, receptor type R (PTPRR), RAS guanyl releasing protein 1 (RASGRP1), transforming growth factor beta receptor I (TGFBR1) and TP53. The correlation analysis conducted with the entire population further suggested that let-7e-5p expression was negatively correlated with ATF2 (r = -0.322, p = 0.049), CASP3 (r = -0.417, p = 0.020), FGFR2 (r = -0.427, p = 0.015), NLK (r = -0.527, p = 0.003), PTPN7 (r = -0.425, p = 0.009), RASGRP1 (r = -0.515, p = 0.004) and TGFBR1 (r = -0.496, p = 0.003). The other genes showed no significant correlation with let-7e-5p expression, as shown in Table 5.

**Table 5:** Correlation analysis of let-7e-5p and gene expression.

Genes*	r †	*P* value
ATF2	-0.322	0.049
CACNB4	0.019	0.913
CACNG4	0.015	0.928
CASP3	-0.417	0.020
CDC25B	-0.293	0.093
ELK4	-0.268	0.177
FGFR2	-0.427	0.015
MAP2K7	-0.151	0.416
MAP3K1	-0.129	0.497
MAP3K2	-0.351	0.053
MAP4K3	0.041	0.807
MAP4K4	-0.216	0.193
MRAS	-0.173	0.299
NGF	-0.161	0.423
NLK	-0.527	0.003
NRAS	-0.149	0.415
PAK1	-0.211	0.272
PDGFB	-0.087	0.604
PTPN7	-0.425	0.009
PTPRR	-0.247	0.146
RASGRP1	-0.515	0.004
TGFBR1	-0.496	0.003
TP53	-0.286	0.081

*The expression of genes is noted as –ΔCt (Ct beta-actin−Ct _gene_).

^†^The Pearson correlation coefficient (r) between the expression level of each candidate gene and let-7e-5p.

## Discussion

In this study, we found that the whole blood level of let-7e-5p was significantly higher in patients with ischemic stroke than in control patients in a Chinese population, and a higher level of let-7e-5p was associated with increased incidence of ischemic stroke. ROC curve and reclassification analyses suggested that the addition of let-7e-5p to traditional risk factors improved the predictive value of ischemic stroke. Bioinformatics prediction and target gene expression analysis showed that let-7e-5p may be involved in the pathogenesis of ischemic stroke by regulating CASP3 and NLK expression, two genes enriched in the MAPK signaling pathway. Collectively, our study demonstrated that let-7e-5p may act as a novel biomarker for ischemic stroke, the biological function of which warrants further investigation.

A biomarker should be non-invasive, easy to identify, and have good sensitivity and specificity. Plasma/serum miRNAs have been extensively studied and have provided a series of excellent biomarkers for the clinical diagnosis or prognosis of disease. However, these biomarkers also face technical limitations because of the extremely low concentrations of circulating miRNAs in the plasma or serum, and some miRNAs with low expression are not detectable. In addition, the absence of ribosomal RNAs in these samples makes assessment of RNA quality difficult. This is also an obstacle to the standardization and translation of the use of plasma/serum miRNAs in a clinical context. Seeking to circumvent this problem, we hypothesized that miRNAs from peripheral blood could be used. Differentially expressed miRNAs in the whole blood of patients with coronary heart disease [19,20] or peripheral arterial disease [21] have been documented. In this study, we demonstrated that the whole blood level of let-7e-5p was significantly higher in ischemic stroke patients and was independently associated with an increased risk of the disease after adjusting for conventional risk factors. The diagnosis potential of let-7e-5p was then evaluated by ROC and reclassification analyses. The addition of let-7e-5p to the traditional risk factor model increased the AUC from 0.74 to 0.82 (p<0.05). Furthermore, let-7e-5p was able to reclassify a significant portion of patients, with an NRI of 16.76% and IDI of 0.10; these results indicate good diagnostic values. Moreover, the correlation between let-7e-5p expression and the platelet parameters indicates the involvement of let-7e-5p in dysfunctional platelets. Our study was the first to demonstrate that the expression level of let-7e-5p in whole blood may serve as a useful noninvasive circulating biomarker for ischemic stroke. While we were preparing this manuscript, a study was published reporting that the expression levels of let-7e-5p in the serum and cerebral spinal fluid of acute ischemic stroke patients were also significantly higher than in the control subjects and showed a specificity as high as 73.4% and a sensitivity of 82.8% in ischemic stroke patients at the acute stage [22]. In addition, it has been suggested that the peripheral blood level of let-7e-5p exhibits good diagnostic value for peripheral arterial disease [21]. In addition, the plasma level of let-7e-5p was higher in hypertension patients than in control subjects [15,23]. In summary, the limited number of clinical studies about let-7e-5p have provided evidence that in addition to being a biomarker for ischemic stroke, let-7e-5p may also act as a molecular biomarker for other cardiovascular diseases. These findings suggest the involvement of let-7e-5p in the cardiovascular disease-related pathomechanism.

Few studies have reported on the biological function of miRNA let-7e-5p. One study demonstrated that let-7e may protect PC12 cells, a nerve cell line, against apoptosis following anoxia/reoxygenation injury by negatively regulating the expression ofCASP3[24]. The involvement of let-7e-5p in immunity has been documented by Quinn et al., who reported that let-7e-5p could regulate the TLR4 signaling pathway during the development of endotoxin tolerance at the receptor, signalling pathway, and gene transcription and translational levels [25]. In addition, Androulidaki et al. also demonstrated that let-7e-5p could repress TLR4, which is a critical protein for lipopolysaccharide-driven TLR signaling, and could regulate endotoxin sensitivity and tolerance [16]. Collectively, these studies only provide limited information about the functional role of let-7e-5p in cardiovascular diseases. In this study, we predicted target genes of let-7e-5p using bioinformatics tools and then tested the miRNA-gene regulation using cell transfection experiments and gene expression analysis of human whole blood samples. We found that two genes, CASP3 and NLK, were likely regulated by let-7e-5p. In cells transfected with let-7e-5p mimics, the mRNA levels of CASP3 and NLK were both significantly lower than in negative control. In addition, in human whole blood samples, the mRNA levels of both CASP3 and NLK were lower in ischemic stroke patients than in control subjects, and they were both negatively correlated with let-7e-5p expression. Our results were consistent with those of a previous study showing the regulation relationship between let-7e-5p and CASP3 [24]; however, this is the first report of the regulation of the gene NLK by let-7e-5p. NLK is an evolutionarily conserved MAP kinase-related kinase and plays a role in multiple processes due to its capacity to regulate a diverse array of signaling pathways, including the Wnt/β-catenin, Activin, IL-6, and Notch signaling pathways [26]. A previous study also demonstrated that NLK was involved in neuronal apoptosis after traumatic brain injury [27]. Therefore, we propose that the differentially expressed let-7e-5p may be functionally relevant to the deregulated apoptosis state by targeting genes CASP3 and NLK. Further investigation is required to confirm the target binding sites by luciferase reporter gene experiments and to evaluate the biological consequences of the artificial disturbance of let-7e-5p expression or the expression of CASP3 and NLK genes.

This study has several strengths. First, the expression levels of let-7e-5p were replicated in two independent case-control studies, and the large sample size provides sufficient statistical power for multivariate analysis. Second, we tested miRNA-gene regulation by conducting cell transfection experiments and miRNA-gene expression correlation analysis in human blood samples, the results of which provide a plausible biological explanation for the epidemiological findings in our study. However, the limitations should also be addressed. First, because this is a case-control study, potential selection bias may influence the interpretation of the results. Second, because circulating miRNAs are often correlated with each other and the combined miRNA panels may provide better sensitivity and specificity for diagnosis, as mentioned by previous studies [28,29], more miRNAs need to be detected to identify miRNA profiles with better prediction efficiency. Third, further investigations are needed to locate the binding sites between let-7e-5p and the target genes identified in this study. The biological consequences for the cerebrovascular diseases following changes in miRNA or target gene expression also merit further investigation. Finally, the causal relationship between the change of let-7e-5p expression and the occurrence of stroke also merits further investigation in a prospective cohort study.

In conclusion, this study suggests that the blood level of let-7e-5p is significantly higher in ischemic stroke patients and is associated with the occurrence of ischemic stroke. Moreover, let-7e-5p regulates the expression of CASP3 and NLK and may be involved in the pathogenesis of ischemic stroke. Further investigations are needed to explore the biological relevance of our findings.

## Supporting Information

S1 Fig The expression levels of let-7e-5p in ischemic stroke patients (n = 20) and control subjects (n = 20). The relative expression levels were normalized to U6 and then log-transformed. The data are expressed as mean±SD.
(DOC)

S1 Table Real-time PCR primer sequences for target genes. (DOC)
