# Supplementary material for: Identification of Blood Let-7e-5p as a Biomarker for Ischemic Stroke
Source: PLoS One. 2016 Oct 24;11(10):e0163951. doi: 10.1371/journal.pone.0163951 (PMC5077157; doi:10.1371/journal.pone.0163951)
Supplement: S1 Fig — The relative expression levels were normalized to U6 and then log-transformed. The data are expressed as mean±SD. (DOC) [file pone.0163951.s001.doc]

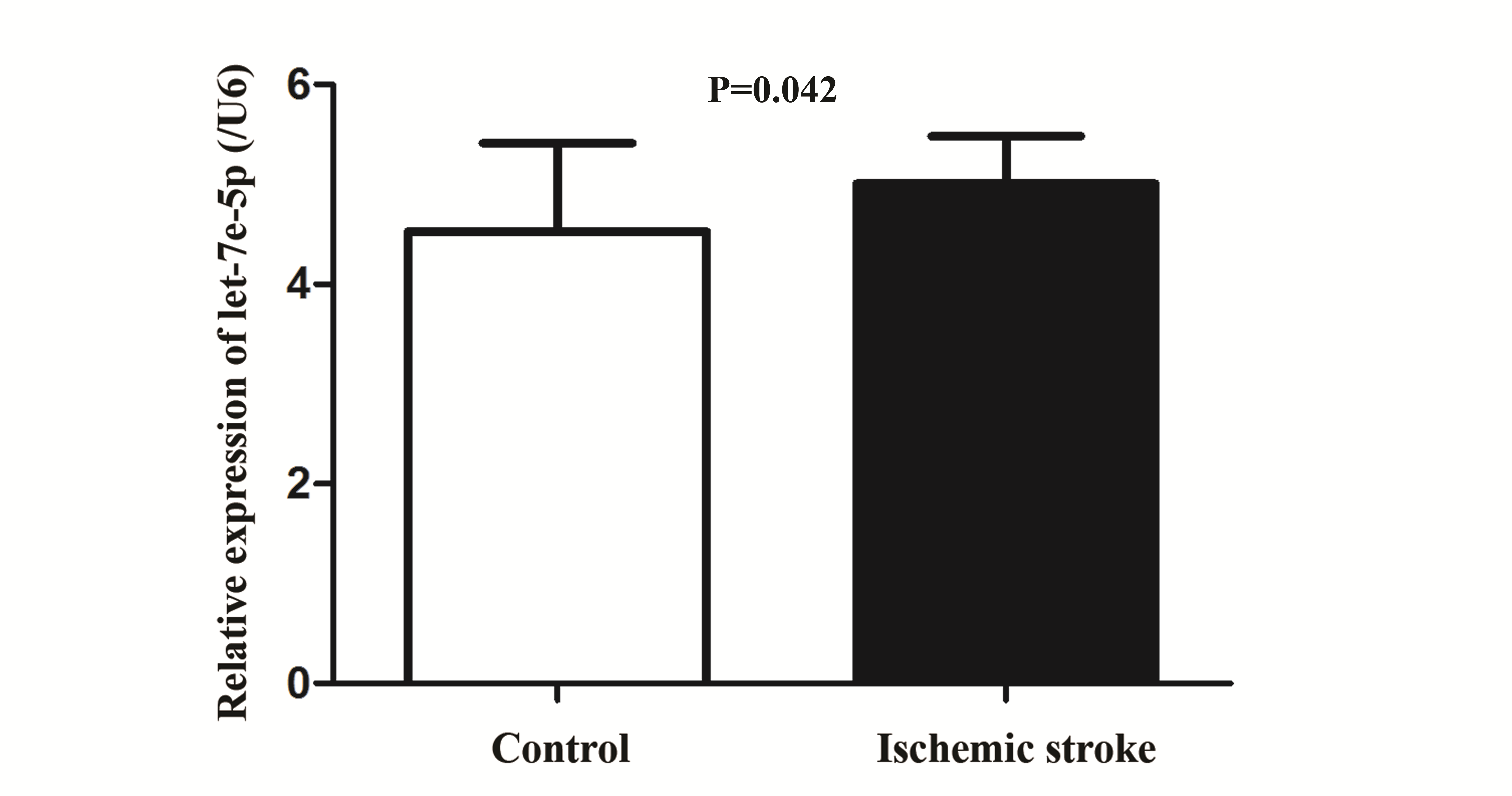


**S1 Fig. The expression levels of let-7e-5p in the ischemic stroke patients (n=20) and control subjects (n=20).** The relative expression levels were normalized to U6 and then log-transformed, expressed as mean±SD.
