# Supplementary material for: Identification of Blood Let-7e-5p as a Biomarker for Ischemic Stroke
Source: PLoS One. 2016 Oct 24;11(10):e0163951. doi: 10.1371/journal.pone.0163951 (PMC5077157; doi:10.1371/journal.pone.0163951)
Supplement: S1 Table — (DOC) [file pone.0163951.s002.doc]

**S1 Table . Real-time PCR primer sequences for target genes.**

| **Gene** | **Abbreviation** | **Forward Primers（5’ →3’）** | **Reverse Primers（5’ →3’）** |
| --- | --- | --- | --- |
| ATF2 | activating transcription factor 2 | AATTGAGGAGCCTTCTGTTGTAG | CATCACTGGTAGTAGACTCTGGG |
| CACNB4 | calcium voltage-gated channel auxiliary subunit beta 4 | GCTGACCTGGTGAAGTGACA | TCCGCTGAACCAGCCATAAA |
| CACNG4 | calcium voltage-gated channel auxiliary subunit gamma 4 | CAAGGCCCCGAGACTTTTCT | TGGCGGATGGAGAGGAACTA |
| CASP3 | caspase 3 | CATGGAAGCGAATCAATGGACT | CTGTACCAGACCGAGATGTCA |
| CDC25B | cell division cycle 25B | CAGCGACTTGCTGCTCAAAA | CGACAGGGAGGATTCGGATG |
| ELK4 | ETS transcription factor | TGGGGAAGAGCAGAGTTCATT | AGACGAGTTTAACCGGTGGG |
| FGFR2 | fibroblast growth factor receptor 2 | AGCACCATACTGGACCAACAC | GGCAGCGAAACTTGACAGTG |
| MAP2K7 | mitogen-activated protein kinase kinase 7 | CCACGTCATTGCCGTTAAGC | GCACGATGTAGGGGCAGTC |
| MAP3K1 | mitogen-activated protein kinase kinase kinase 1 | CATCAGGTCGCACAGTGAAAT | TCAGGGCTATATGGTGAGAAGC |
| MAP3K2 | mitogen-activated protein kinase kinase kinase 2 | CCCCAGGTTACATTCCAGATGA | GCATTCGTGATTTTGGATAGCTC |
| MAP4K3 | mitogen-activated protein kinase kinase kinase kinase 3 | CAGCTGTTGAGAGGAAGGGG | CAGCAACAAGAGGCTCAGGA |
| MAP4K4 | mitogen-activated protein kinase kinase kinase kinase 4 | AGGCCATGTTACTGGAGTGC | TTGTTGCAACTGCCTCTGGA |
| MRAS | muscle RAS oncogene homolog | GCGTCAAAGACAGGGAGTCA | GGTCCTTGGCACTGGTTTCT |
| NGF | nerve growth factor | CCAGCCACAGCAGAGTTTTGG | GAGTGTGGTTCCGCCTGTAT |
| NLK | nemo like kinase | CGCAAAAATGATGGCGGCTTA | CCCAGGGTTTAACATGGCTG |
| NRAS | neuroblastoma RAS viral oncogene homolog | ATGACTGAGTACAAACTGGTGGT | CATGTATTGGTCTCTCATGGCAC |
| PAK1 | p21 (RAC1) activated kinase 1 | CAGCCCCTCCGATGAGAAATA | CAAAACCGACATGAATTGTGTGT |
| PDGFB | platelet derived growth factor subunit B | CAGCGCCCATTTTTCATTCC | TTTTCTCTTTGCAGCGAGGC |
| PTPN7 | protein tyrosine phosphatase, non-receptor type 7 | GGGAGGTCACCCTACACTTTC | TGGTCTTGTATCGGTCCTTGG |
| PTPRR | protein tyrosine phosphatase, receptor type R | ACCTATCGCCCATCACATTACA | GCGGTGGTAGCTTTGATCTCA |
| RASGRP1 | RAS guanyl releasing protein 1 | ACATCACCCAGTTCCGAATGA | GCTGTCAATGAGATCGTCCAG |
| TGFBR1 | transforming growth factor beta receptor 1 | GCTGTATTGCAGACTTAGGACTG | TTTTTGTTCCCACTCTGTGGTT |
| TP53 | tumor protein p53 | CAGCACATGACGGAGGTTGT | TCATCCAAATACTCCACACGC |
